# Artificial intelligence-aided clinical annotation of a large multi-cancer genomic dataset

**DOI:** 10.1038/s41467-021-27358-6

**Published:** 2021-12-15

**Authors:** Kenneth L. Kehl, Wenxin Xu, Alexander Gusev, Ziad Bakouny, Toni K. Choueiri, Irbaz Bin Riaz, Haitham Elmarakeby, Eliezer M. Van Allen, Deborah Schrag

**Affiliations:** 1 From Dana-Farber Cancer Institute, Boston, MA USA; 2 Brigham and Women’s Hospital, Boston, MA USA; 3 Harvard Medical School, Boston, MA USA; 4 Mayo Clinic, Rochester, USA; 5 The Broad Institute, Rochester, USA; 6 Memorial-Sloan Kettering Cancer Center, New York, USA

**Keywords:** Cancer, Molecular medicine

## Abstract

To accelerate cancer research that correlates biomarkers with clinical endpoints, methods are needed to ascertain outcomes from electronic health records at scale. Here, we train deep natural language processing (NLP) models to extract outcomes for participants with any of 7 solid tumors in a precision oncology study. Outcomes are extracted from 305,151 imaging reports for 13,130 patients and 233,517 oncologist notes for 13,511 patients, including patients with 6 additional cancer types. NLP models recapitulate outcome annotation from these documents, including the presence of cancer, progression/worsening, response/improvement, and metastases, with excellent discrimination (AUROC > 0.90). Models generalize to cancers excluded from training and yield outcomes correlated with survival. Among patients receiving checkpoint inhibitors, we confirm that high tumor mutation burden is associated with superior progression-free survival ascertained using NLP. Here, we show that deep NLP can accelerate annotation of molecular cancer datasets with clinically meaningful endpoints to facilitate discovery.

## Abstract (web summary)

To accelerate cancer research that correlates biomarkers with clinical endpoints, methods are needed to ascertain outcomes from electronic health records at scale. Here, the authors train natural language processing to extract outcomes for participants in a precision oncology study.

## Introduction

Modern cancer research increasingly focuses on precision oncology^1^, seeking to identify prognostic and predictive biomarkers to guide drug discovery and selection of optimal therapies for individual patients. Pursuing this objective, particularly for uncommon cancers or rare biomarker patterns for common malignancies, requires large datasets of tumors that have undergone deep molecular characterization. Such datasets are increasingly available^2–5^, but their utility has been limited by the absence of scalable methods for gathering the clinical outcomes data necessary to pursue patient-relevant research questions. Basic outcomes, such as whether and when a cancer progresses or responds to treatment, are generally not recorded in a structured format outside of therapeutic clinical trials. Extraction of such outcomes from electronic health records has historically required resource-intensive manual medical records review, which has been further limited by the absence of a standardized data model for medical record annotation across studies.

We have developed the structured Pathology, Radiology/Imaging, Signs/Symptoms, Medical oncologist assessment, and bioMarkers (PRISSMM) data model for extracting clnical outcomes for linkage to genomic datasets in a structured and reproducible manner^6^. PRISSMM provides a rubric for manual abstraction of specific cancer outcomes from individual imaging reports and medical oncologist notes. These outcomes include the presence of cancer within specific documents and at specific body sites; cancer progression/worsening; and cancer response/improvement. Annotations of individual reports along the disease trajectory can then be analyzed to derive relevant endpoints, such as progression-free survival indexed from initiation of a given treatment^7^.

The PRISSMM annotations generated for individual electronic health record (EHR) documents can also be leveraged as labels to train machine learning models to perform annotation automatically. We previously demonstrated the feasibility of training interpretable natural language processing (NLP) models to extract outcomes from imaging reports^8^ and medical oncologist notes^9^ for patients with non-small cell lung cancer. The generalizability of this approach to other types of cancer and its application to create a linked clinico-genomic dataset have not been previously described.

Here, we train such models using labeled data from patients with multiple types of cancer; demonstrate their generalizability to cancer types not seen in training; and evaluate associations between NLP-derived clinical annotations and overall survival. Finally, we create a large multi-cancer clinico-genomic dataset by applying this technique to EHR data at scale, and we demonstrate the utility of this type of dataset by exploring associations between tumor mutation burden and progression-free survival on immune checkpoint inhibitor therapy.

## Results

### Cohort

We identified patients with any of 13 common malignant solid tumors whose tumor specimens underwent next generation sequencing (NGS) through the PROFILE initiative at Dana-Farber Cancer Institute (DFCI) from 2013 to 2021^4,10^. Cancer types included breast, colorectal, endometrial, gastric/esophageal, head and neck, leiomyoscarcoma, non-small cell lung, melanoma, high-grade serous ovarian, pancreatic, prostate, renal cell, and urothelial cancers. The imaging report cohort included 13,130 patients with 304,160 reports (Table 1), and the oncologist note cohort included 13,511 patients with 232,575 reports (Table 2); most patients (*n* = 11,096) had both imaging reports and oncologist notes. Manual medical record review per the PRISSMM framework was performed for a subset of patients with non-small cell lung, breast, colorectal, pancreatic, prostate, renal cell, or urothelial cancer. This annotation included 2,830 patients with 31,196 labeled imaging reports (Table 1) and 2,747 patients with 32,311 labeled medical oncologist notes (Table 2). No manual annotation was performed for patients with endometrial, gastric/esophageal, head and neck, leiomyosarcoma, melanoma, or high-grade serous ovarian cancers. Additional details about the cohort, including the distribution of common somatic mutations on NGS, are provided in Tables 1 and 2.

**Table 1:** Characteristics of patients with radiology reports for analysis.

	Total number of patients and radiology reports		Number of patients with unlabeled radiology reports and number of unlabeled radiology reports		Number of patients with labeled radiology reports and # of labeled radiology reports	
	Patients *N* (%)	Reports *N* (%)	Patients *N* (%)	Reports *N* (%)	Patients *N* (%)	Reports *N* (%)
Total cohort	13130 (100)	304160 (100)	10300 (100)	272964 (100)	2830 (100)	31196 (100)
Sex						
Male	5621 (43)	105503 (35)	4055 (39)	89849 (33)	1566 (55)	15654 (50)
Female	7509 (57)	198657 (65)	6245 (61)	183115 (67)	1264 (45)	15542 (50)
Age at next generation genomic sequencing						
<40	625 (5)	14439 (5)	488 (5)	12835 (5)	137 (5)	1604 (5)
40–49	1329 (10)	30868 (10)	999 (10)	26490 (10)	330 (12)	4378 (14)
50–59	3092 (24)	75681 (25)	2400 (23)	67920 (25)	692 (24)	7761 (25)
60–69	4172 (32)	99399 (33)	3295 (32)	90158 (33)	877 (31)	9241 (30)
70–79	2944 (22)	65229 (21)	2335 (23)	58700 (22)	609 (22)	6529 (21)
80+	968 (7)	18544 (6)	783 (8)	16861 (6)	185 (7)	1683 (5)
Race as recorded in the electronic health record						
Asian	424 (3)	10724 (4)	353 (3)	9716 (4)	71 (3)	1008 (3)
African-American	458 (3)	10649 (4)	348 (3)	9470 (3)	110 (4)	1179 (4)
Native American	11 (<1)	193 (<1)	10 (<1)	184 (<1)	1 (<1)	9 (<1)
Pacific Islander	4 (<1)	144 (<1)	4 (<1)	144 (<1)	0 (0)	0 (0)
White	11760 (90)	272156 (89)	9205 (89)	244173 (89)	2555 (90)	27983 (90)
More than one race	39 (<1)	729 (<1)	33 (<1)	652 (<1)	6 (<1)	77 (<1)
Other/unknown	434 (3)	9565 (3)	347 (3)	8625 (3)	87 (3)	940 (3)
Cancer type						
Breast	2029 (15)	63789 (21)	1676 (16)	58209 (21)	352 (12)	5527 (18)
Colorectal	1958 (15)	37570 (12)	1493 (14)	32986 (12)	466 (16)	4588 (15)
Endometrial	482 (4)	9801 (3)	482 (5)	9801 (4)	0 (0)	0 (0)
Gastroesophageal	878 (7)	19794 (7)	878 (9)	19794 (7)	0 (0)	0 (0)
Head and neck	461 (4)	8796 (3)	460 (4)	8795 (3)	0 (0)	0 (0)
Leiomyosarcoma	144 (1)	6241 (2)	144 (1)	6241 (2)	0 (0)	0 (0)
Non-small cell lung	3378 (26)	82609 (27)	2763 (27)	73758 (27)	614 (22)	8838 (28)
Melanoma	733 (6)	20621 (7)	731 (7)	20591 (8)	0 (0)	0 (0)
Ovarian	646 (5)	22248 (7)	646 (6)	22248 (8)	0 (0)	0 (0)
Pancreatic	685 (5)	7854 (3)	295 (3)	4477 (2)	394 (14)	3450 (11)
Prostate	617 (5)	7506 (2)	164 (2)	2851 (1)	453 (16)	4676 (15)
Renal cell carcinoma	499 (4)	4737 (2)	84 (<1)	1721 (<1)	415 (15)	3016 (10)
Urothelial carcinoma	620 (5)	12594 (4)	484 (5)	11492 (4)	136 (5)	1101 (4)
Common tumor genomic variants						
TP53 mutation	5330 (41)	124663 (41)	2486 (42)	112237 (41)	1044 (37)	12426 (40)
KRAS mutation	2785 (21)	53735 (18)	2012 (20)	45775 (17)	773 (27)	7960 (26)
PIK3CA mutation	1738 (13)	43168 (14)	1455 (14)	39788 (15)	283 (10)	3380 (11)
APC mutation	1215 (9)	24381 (8)	942 (9)	21627 (8)	273 (10)	2754 (9)
BRAF mutation	688 (5)	15938 (5)	587 (6)	14918 (5)	101 (4)	1020 (3)

**Table 2:** Characteristics of patients with medical oncologist notes for analysis.

	Total number of patients and medical oncologist notes		Number of patients with unlabeled medical oncology notes and number of unlabeled medical oncology notes		Number of patients with labeled medical oncology notes and number of labeled medical oncology notes	
	Patients *N* (%)	Reports *N* (%)	Patients *N* (%)	Reports *N* (%)	Patients *N* (%)	Reports *N* (%)
Total cohort	13511 (100)	232575 (100)	10764 (100)	200264 (100)	2747 (100)	32311 (100)
Sex						
Male	5561 (41)	88755 (38)	4088 (38)	71790 (36)	1473 (54)	16965 (53)
Female	7950 (59)	143820 (62)	6676 (62)	128474 (64)	1274 (46)	15346 (47)
Age at next generation tumor genomic sequencing						
<40	733 (5)	13111 (6)	574 (5)	11226 (6)	159 (6)	1885 (6)
40–49	1477 (11)	26420 (11)	1139 (11)	21511 (11)	338 (12)	4909 (15)
50–59	3297 (24)	61142 (26)	2616 (24)	52618 (26)	681 (25)	8524 (26)
60–69	4277 (32)	74818 (32)	3432 (32)	65689 (33)	845 (31)	9129 (28)
70–79	2864 (21)	45914 (20)	2301 (21)	39553 (20)	563 (20)	6361 (20)
80+	863 (6)	11170 (5)	702 (7)	9667 (5)	161 (6)	1503 (5)
Race as recorded in the electronic health record						
Asian	439 (3)	7914 (3)	361 (3)	6902 (3)	78 (3)	1012 (3)
African-American	445 (3)	7785 (3)	344 (3)	6550 (3)	101 (4)	1235 (4)
Native American	13 (<1)	99 (<1)	11 (<1)	93 (<1)	2 (<1)	6 (<1)
Pacific Islander	4 (<1)	123 (<1)	4 (<1)	123 (<1)	0 (0)	0 (0)
White	12132 (90)	207897 (89)	9653 (90)	179147 (89)	2479 (90)	28750 (89)
More than one race	36 (<1)	738 (<1)	31 (<1)	655 (<1)	5 (<1)	83 (<1)
Other/unknown	442 (3)	8019 (3)	360 (3)	6794 (3)	82 (3)	1225 (4)
Cancer type						
Breast	2382 (18)	47595 (20)	1972 (18)	41462 (21)	409 (15)	6105 (19)
Colorectal	2447 (18)	35459 (15)	1922 (18)	29451 (15)	526 (19)	6011 (19)
Endometrial	524 (4)	6754 (3)	524 (5)	6754 (3)	0 (0)	0 (0)
Gastroesophageal	1019 (8)	16363 (7)	1019 (9)	16363 (8)	0 (0)	0 (0)
Head and neck	447 (3)	10901 (5)	446 (4)	10898 (5)	0 (0)	0 (0)
Leiomyosarcoma	168 (1)	4581 (2)	168 (2)	4581 (2)	0 (0)	0 (0)
Non-small cell lung	2838 (21)	43360 (19)	2297 (21)	38090 (19)	540 (20)	5237 (16)
Melanoma	756 (6)	19100 (8)	754 (7)	19064 (10)	0 (0)	0 (0)
Ovarian	713 (5)	19885 (9)	713 (7)	19885 (10)	0 (0)	0 (0)
Pancreatic	878 (6)	9111 (4)	397 (4)	5016 (3)	485 (18)	4173 (13)
Prostate	549 (4)	7818 (3)	99 (<1)	1678 (8)	451 (16)	6167 (19)
Renal cell carcinoma	364 (3)	4434 (2)	93 (<1)	756 (<1)	271 (10)	3680 (11)
Urothelial carcinoma	426 (3)	7214 (3)	360 (3)	6266 (3)	65 (2)	938 (3)
Common tumor genomic variants						
TP53 mutation	5780 (43)	99351 (43)	4675 (43)	87185 (44)	1105 (40)	12166 (38)
KRAS mutation	2993 (22)	39485 (17)	2161 (20)	31814 (16)	832 (30)	7671 (24)
PIK3CA mutation	1897 (14)	32896 (14)	1618 (15)	29345 (15)	279 (10)	3551 (11)
APC mutation	1457 (11)	22278 (10)	1147 (11)	18628 (9)	310 (11)	3650 (11)
BRAF mutation	727 (5)	13091 (6)	628 (6)	12071 (6)	99 (4)	1020 (3)

### NLP model performance on a per-document basis

When evaluated in the test subset, NLP models trained to extract outcomes from all labeled training imaging reports yielded areas under the receiver operating characteristic curve (AUROCs) of 0.98 for the any-cancer outcome, 0.95 for progression/worsening cancer, and 0.97 for response/improving cancer. NLP models also reliably ascertained the presence of cancer at specific metastatic sites, with AUCs of 0.99 for brain, 0.99 for bone, 0.99 for adrenal, 0.99 for liver, 0.98 for lung, 0.98 for nodal, and 0.99 for peritoneal metastasis (Table 3). The sensitivity, specificity, positive predictive value (PPV), and negative predictive value (NPV) of these models at both the best F1 and best Youden cutoff thresholds are provided in Supplementary Fig. 1A–J. Performance was consistent (AUROCs > 0.90) across types of cancer, with the exception of peritoneal metastasis for urothelial carcinoma (AUROC 0.81). Model discrimination remained similar across individual cancer types even when models were re-trained without data for that cancer type (for example, AUROC range 0.95–0.98 for the any-cancer outcome, 0.92–0.96 for the progression/worsening outcome, and 0.94–0.98 for the response/improvement outcome; Table 3).

**Table 3:** Areas under the receiver operating characteristic curve (AUROCs) for NLP models that interpret imaging report text to ascertain clinical outcomes, as evaluated in the labeled test set.

Clinical outcome	Models trained on imaging reports for patients with all listed cancer types							
	All patients	Breast cancer	Colorectal cancer	NSCLC	Pancreatic cancer	Prostate cancer	Renal cell carcinoma	Urothelial carcinoma
Any cancer	0.98	0.98	0.98	0.97	0.97	0.98	0.97	0.97
Progression	0.95	0.95	0.96	0.96	0.93	0.96	0.96	0.94
Response	0.97	0.98	0.99	0.96	0.97	0.94	0.97	0.99
Brain metastasis	0.99	0.97	1.0	0.99	1.0	0.98	0.97	*
Bone metastasis	0.99	0.98	0.99	0.98	0.99	0.98	0.99	0.99
Adrenal metastasis	0.99	0.99	0.99	1.0	1.0	*	0.95	0.99
Liver metastasis	0.99	1.0	0.99	1.0	0.97	0.98	1.0	1.0
Lung metastasis	0.98	0.99	0.99	0.97	0.98	0.97	0.98	0.99
Nodal metastasis	0.98	0.99	0.97	0.98	0.96	0.99	0.99	0.97
Peritoneal metastasis	0.99	0.99	0.99	1.0	0.96	1.0	*	0.81
Models trained on imaging reports for patients with all cancers except for the type under evaluation								
Any cancer	†	0.98	0.98	0.95	0.95	0.98	0.98	0.96
Progression	†	0.94	0.96	0.96	0.92	0.96	0.95	0.94
Response	†	0.98	0.99	0.96	0.97	0.94	0.96	0.99
Brain metastasis	†	0.97	1.0	0.99	1.0	0.98	0.99	*
Bone metastasis	†	0.97	0.98	0.98	0.99	0.98	0.99	1.0
Adrenal metastasis	†	0.99	0.99	0.99	1.0	*	0.94	0.99
Liver metastasis	†	1.0	0.99	0.99	0.96	0.98	1.0	1.0
Lung metastasis	†	0.99	0.99	0.94	0.97	0.97	0.97	0.99
Nodal metastasis	†	0.99	0.98	0.98	0.96	0.99	0.99	0.97
Peritoneal metastasis	†	0.99	0.98	1.0	0.97	1.0	*	0.77

*Not enough variation in outcome label to evaluate AUROC

†Not applicable

Models trained to extract outcomes from all labeled training medical oncologist notes yielded AUROCs of 0.93 for the any-cancer outcome, 0.92 for the progression/worsening outcome, and 0.93 for the response/improving outcome (Table 4). The sensitivity, specificity, PPV, and NPV of these models at the best F1 and best Youden cutoff thresholds are provided in Supplementary Fig. 1K–M. Performance was generally consistent (AUROCs > 0.90) across cancer types, except for AUROCs of 0.78 for the any-cancer outcome in pancreas cancer; 0.87 for cancer progression and response in prostate cancer; 0.86 for progression and 0.89 for response in renal cell carcinoma; and 0.78 for progression in urothelial cancer (Table 4). As observed for the imaging reports, performance for medical oncologist note evaluation was similar when evaluated using labels from cancer types excluded from training; Table 4.

**Table 4:** Areas under the receiver operating characteristic curve (AUROCs) for NLP models that interpret medical oncologist progress note text to ascertain clinical outcomes, as evaluated in the labeled test set.

Clinical outcome	Models trained on all cancer types							
	All patients	Breast cancer	Colorectal cancer	NSCLC	Pancreatic cancer	Prostate cancer	Renal cell carcinoma	Urothelial carcinoma
Any cancer	0.93	0.98	0.98	0.95	0.78	0.91	0.91	0.95
Progression	0.92	0.97	0.91	0.96	0.92	0.87	0.86	0.78
Response	0.93	0.96	0.95	0.95	0.91	0.87	0.89	0.99
Models trained on all cancers except for the type under evaluation								
Any cancer	†	0.98	0.97	0.95	0.79	0.85	0.90	0.95
Progression	†	0.96	0.90	0.95	0.91	0.84	0.84	0.78
Response	†	0.94	0.94	0.94	0.88	0.83	0.88	1.0

†Not applicable

Given variability by cancer type in model performance for medical oncologist notes, we explored apparent incorrect model predictions for the any-cancer outcome for pancreatic cancer. For pancreatic cancer, there were 467 notes for 48 patients in the test set. Of these, 45 notes for 7 patients were apparent false positives: that is, the predicted probability of “any cancer” was above the best F1 threshold, but the manual annotation indicated no cancer in that note. Of the 45 notes, 28 notes belonged to one patient who had a history of pancreatic cancer in remission but also had metastatic breast cancer treated after the index pancreatic cancer. Most of these notes corresponded to a time period when the breast cancer was under active treatment but the pancreatic cancer was in remission. Model predictions appeared to capture this active (breast) cancer period, but manual annotations indicated the absence of the specific index pancreatic cancer that led to cohort eligibility for manual abstraction. If this single patient’s notes had been removed from the full test set, the calculated AUROC would have increased from 0.78 to 0.93. Overall, this review of “false positives” indicates that apparent NLP model errors may consist of a mixture of outcomes truly missed by trained models, and cases in which the models may have captured the intended outcome but the manual annotations used for evaluation did not.

In a sensitivity analysis performed to evaluate the generalizability of these NLP models when applied for inference to future time periods, models for imaging reports and oncologist notes were re-trained using reports through 2017 but evaluated using reports from 2018. AUROCs remained ≥0.90 for all endpoints (Supplementary Table 1).

### Associations between AI-curated endpoints and overall survival

Among 4,953 patients in the imaging report cohort with any of the 13 types of cancer in this study who received palliative-intent systemic therapy, progression/worsening, treated as a time-varying covariate, was associated with increased mortality (Hazard ratio, HR, 2.03, 95% CI 1.87–2.20); and response/improvement, treated as a time-varying covariate, was associated with decreased mortality (HR 0.36, 95% CI 0.30–0.43). These associations remained consistent in an analysis restricted to the subgroup of 1,241 patients who had any of the 6 cancer types for which no manually labeled documents were available (HR for progression/worsening, 1.84, 95% CI 1.56–2.16; HR for response/improvement, 0.27, 95% CI 0.18–0.41); Table 5. Among 5,064 patients in the medical oncologist note cohort with any of the 13 cancer types in this study who received palliative-intent systemic therapy, progression/worsening was associated with increased mortality (HR 4.34, 95% CI 4.02–4.70), and response/improvement was associated with decreased mortality (HR 0.45, 95% CI 0.38–0.54). Again, these associations remained consistent in the subgroup of 1,267 patients with any of the 6 cancer types for which no manually labeled documents were available (HR for progression/worsening, 4.68, 95% CI 3.99–5.48; HR for response/improvement, 0.46, 95% CI 0.30–0.70); Table 5. Associations between these outcomes and OS in individual cancer types are also provided in Table 5.

**Table 5:** Association between PRISSMM outcomes and overall survival among patients receiving palliative-intent systemic therapy (Hazard ratio, 95% confidence interval).

Cohort	PRISSMM imaging report annotations derived from natural language processing models			PRISSMM medical oncologist note annotations derived from natural language processing models		
	N	Progression/worsening	Response/Improvement	*N*	Progression/worsening	Response/Improvement
All patients*	4953	2.03 (1.87–2.20)	0.36 (0.30–0.43)	5064	4.34 (4.02–4.70)	0.45 (0.38–0.54)
All patients with labeled cancer types*	3712	2.10 (1.91–2.31)	0.39 (0.32–0.47)	3797	4.25 (3.88–4.64)	0.44 (0.37–0.55)
Breast cancer†	1058	2.19 (1.81–2.65)	0.44 (0.30–0.65)	1080	6.30 (5.23–7.58)	0.56 (0.36–0.87)
Colorectal cancer†	674	1.91 (1.54–2.37)	0.19 (0.10–0.36)	701	3.97 (3.24–4.87)	0.27 (0.14–0.55)
NSCLC†	1151	2.25 (1.92–2.64)	0.46 (0.34–0.62)	1141	3.53 (3.02–4.12)	0.37 (0.27–0.52)
Pancreatic cancer†	286	1.82 (1.34–2.47)	0.24 (0.11–0.51)	305	4.13 (3.13–5.45)	0.35 (0.18–0.70)
Prostate cancer†	197	1.74 (1.11–2.74)	0.87 (0.35–2.14)	211	3.41 (2.28–5.10)	0.61 (0.18–2.11)
Renal cell carcinoma†	161	1.91 (1.22–3.00)	0.69 (0.26–1.85)	173	2.95 (2.04–4.26)	0.68 (0.40–1.15)
Urothelial carcinoma†	185	2.04 (1.35–3.09)	0.23 (0.07–0.73)	186	3.85 (2.46–6.03)	0.57 (0.30–1.09)
All patients with unlabeled cancer types*	1241	1.84 (1.56–2.16)	0.27 (0.18–0.41)	1267	4.68 (3.99–5.48)	0.46 (0.30–0.70)
Endometrial	107	1.52 (0.88–2.63)	0.21 (0.06–0.87)	107	6.47 (3.71–11.29)	0.68 (0.17–2.70)
Gastroesophageal cancer	364	2.05 (1.57–2.69)	0.14 (0.06–0.35)	368	5.71 (4.36–7.48)	0.22 (0.08–0.61)
Head and neck cancer	91	1.91 (1.06–3.49)	0.39 (0.13–1.24)	96	2.84 (1.68–4.80)	0.51 (0.16–1.61)
Leiomyosarcoma	72	1.09 (0.57–2.10)	0.14 (0.02–1.17)	72	‡	‡
Melanoma	289	3.58 (2.41–5.31)	0.60 (0.28–1.27)	303	5.69 (3.96–8.16)	0.85 (0.44–1.62)
Ovarian cancer	318	1.13 (0.82–1.55)	0.26 (0.12–0.60)	321	3.16 (2.34–4.27)	0.58 (0.24–1.41)

Hazard ratios derived from multivariable models including all PRISSMM imaging outcomes, which were treated as time-varying covariates. Hazard ratios therefore capture the differential mortality risks associated with time periods following either cancer progression/worsening or cancer response/improvement. Only time following genomic testing was treated as at risk for mortality, since genomic testing was a cohort eligibility criterion. Automated annotations were derived from NLP models using a cross-validation approach, such that inference for each patient was performed using models that excluded that patient from training.

*Analysis also adjusted for cancer type.

†Labeled cancer type (labeled data for this individual cancer type were used to train NLP models).

‡Insufficient data for stable regression estimates.

### Correlations between PRISSMM-derived progression-free survival (PFS) endpoints and OS

Progression-free survival, or the time from an index event to the composite of cancer growth or death, is commonly applied as an outcome in cancer research^11^. It may be considered as a potential endpoint in certain contexts where prolonged survival is common, such that using an overall survival endpoint may require prolonged follow-up to accrue an adequate number of events; and when multiple lines of treatment are common, such that associations between early lines of treatment and outcomes may be masked by subsequent crossover to other treatments. The utility of PFS as a surrogate for OS is sometimes evaluated by measuring its correlation with OS either on a treatment effect or per-patient basis^7,12–14^. Here, we measured the correlation between PFS endpoints derived using our NLP models and OS on a per-patient basis among 5,481 patients in the cohort who initiated palliative-intent therapy before December 31, 2019.

Candidate PFS endpoints for this analysis included PFS-I, or the time to first progression/worsening documented on imaging report, or death; PFS-M, the time to first progression/worsening documented on medical oncologist assessment, or death; PFS-I-or-M, time to first indication of progression/worsening on imaging report or medical oncologist assessment, or death, whichever was earliest; and PFS-I-and-M, time from treatment start to progression/worsening having been documented on both an imaging report and a medical oncologist assessment, or death. Correlations between each of these outcomes and OS were calculated. The highest correlation was between PFS-I-and-M and OS (rho = 0.75, 95% CI 0.73–0.76), which was similar to the correlation we previously observed for manually labeled endpoints for non-small cell lung cancer and colorectal cancer^7^. The correlation between PFS-I and OS was 0.64 (95% CI 0.62–0.66); between PFS-M and OS, 0.59 (95% CI 0.56–0.61); and between PFS-I-or-M and OS, 0.53 (95% CI 0.51–0.55). Kaplan–Meier estimates for OS and each PFS outcome are depicted in Supplementary Fig. 2.

### Association between tumor mutation burden and PFS among patients receiving immunotherapy

**Fig. 1:**
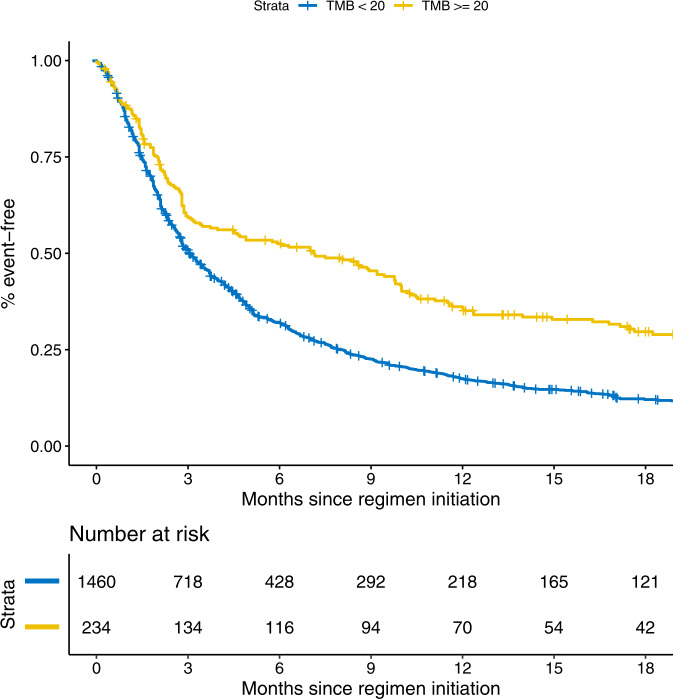
Example of a clinico-genomic analysis based on outcomes ascertained using natural language processing models: Association between TMB and progression-free survival after initation of immunotherapy. High tumor mutational burden defined as >=20 mutations per megabase. Results in this figure represent unadjusted Kaplan-Meier curves. Events were recorded using the “PFS-I-and-M” endpoint, defined as the earlier of death, or the time by which both a medical oncologist note and an imaging report had described cancer progression/worsening. Progression/worsening was defined using natural language processing models applied to imaging reports and medical oncologist notes. Survival curves were not adjusted for left truncation, since progression events were possible prior to genomic testing and cohort eligibility.

To demonstrate an application of a clinico-genomic dataset in which clinical outcomes were defined using our NLP models, we examined the association between PFS and tumor mutation burden (TMB), which has previously been characterized as a predictive biomarker for patients receiving immunotherapy^15–17^, among 1,374 patients who received 1,694 lines of palliative-intent systemic therapy containing an immune checkpoint inhibitor. We defined PFS as PFS-I-and-M, based on the results of the analysis of correlation with OS above. The index time point was initiation of an immunotherapy regimen. In a Cox model that treated TMB as a continuous variable, with adjustment for cancer type and line of therapy and accounting for repeated measures among patients, there was a significant association between a higher TMB and longer survival (HR 0.99, 95% CI 0.98–0.99, per mutation per megabase; *p* < 0.001). This association persisted when TMB was dichotomized into high (>=20 mutations/megabase) or low (<20 mutations/megabase)^15^ (HR for TMB-high, 0.64, 95% CI 0.53–0.78, *p* < 0.001), including among patients with any of the 5 cancer types for which no labeled data were available (HR for TMB-high, 0.60, 95% CI 0.45–0.80, *p* < 0.001). Notably, PFS events accrued more quickly than did mortality events in these analyses. For example, the median PFS was 3.1 months for patients with TMB-low tumors and 7.1 months for patients with TMB-high tumors, whereas the median OS was 11.2 months for patients with TMB-low tumors and 21.6 months for TMB-high tumors, even after left truncation was performed for the OS analyses to remove immortal time related to the requirement that patients have genomic testing for cohort entry^18^. Unadjusted Kaplan–Meier curves depicting associations between TMB and PFS are provided in Fig. 1 and Supplementary Fig. 3.

## Discussion

We deployed deep NLP “artificial intelligence” neural network methods to extract clinical outcomes, including cancer response, progression, and metastatic sites, for linkage to genomic data for a large cohort of patients with common solid tumors. Models for this study were trained and validated using labels based manual review of approximately 21% of the overall cohort, but the models were able to generalize to patients whose records had not been reviewed and even to cancer types other than those included in model training. Automatically extracted outcomes demonstrated expected correlations with overall survival. We demonstrated the utility of this approach for genomically-informed cancer research by measuring the association between tumor mutation burden and progression-free survival across cancer types.

These findings have implications for the conduct of both retrospective and prospective precision oncology research. In contexts where large quantities of molecular data are available, including academic cancer centers^4,10,19,20^ as well as linkages between community practice EHRs and genomic data^15^, limited clinical annotation could be performed to train models that can annotate remaining held-out records, substantially increasing statistical power to understand rare response patterns and uncommon biomarkers. This method could also inform application of electronic health record annotation for ascertaining outcomes in prospective pragmatic trials for which traditional radiographic response assessment might be impractical or prohibitive.

Strengths of this analysis include its application of a structured framework for medical record annotation to derive clinical outcomes data across cancer types and its demonstration that NLP models trained to extract outcomes from records for patients with one type of cancer can generalize to outcome extraction for other cancer types. Limitations include the requirement for at least some manual annotation to train and validate initial models; practical deployment in a prospective context would also require limited periodic annotation to monitor model performance as annotation expands to new cancer types and data distributions shift. The reports selected for manual annotation essentially constituted a convenience sample, since we gathered annotations from separate clinical projects at DFCI that were ongoing using the same PRISSMM framework. However, the generalizability of models trained using this approach to cancer types excluded from training may indicate that this convenience sampling is not prohibitive for creation of a large dataset using NLP, which may be of benefit to health systems seeking to deploy this approach using available resources. Since this is a single-institution dataset, care that patients might receive outside of our health system might not be captured; this is a problem common to any EHR-based analysis.

The NLP models trained in this analysis are probabilistic; they generate continuous output scores that can be interpreted as the predicted probability that a given outcome was present in an EHR text document. No such model is perfect; false positive and false negative predictions will occur at any given threshold probability. Interestingly, when we examined apparent false positive predictions for medical oncologist note models in a cancer type where they appeared to underperform, we found that the apparent model “errors” were a mixture of true errors and cases in which the model predictions may in fact have been more accurate than the manual annotations used to evaluate them. This demonstrates the potential utility of this approach to ascertain clinical outcomes even if those outcomes are to some degree subjective; given a database of generally high-quality labels, useful models can be trained even if the labels are occasionally erroneous or reflect true clinical uncertainty. Such models could even be deployed to assist with quality assurance in ongoing manual annotation efforts.

Further work is needed to evaluate the quantity of manually annotated data necessary to train outcome extraction models with acceptable performance, and to evaluate the generalizability of this approach to other institutions and to clinico-genomic datasets derived from multiple institutions or practices. Notably, however, multi-institutional clinico-genomic data can be collected with this approach via independent annotation and model deployment at each site, without necessarily requiring that the models themselves be shared or generalize across institutions. This may facilitate derivation of such multi-site datasets despite regulatory barriers to sharing protected health information (PHI), especially since there is some concern that PHI might be encoded into neural network model parameters through the training process^21^.

Finally, in a demonstration of clinico-genomic questions that can be asked using a dataset created using AI, we found that higher TMB was associated with superior PFS on immune checkpoint inhibitor therapy in a cohort of patients with multiple types of cancer, consistent with reports from studies that used traditional radiographic progression assessment^17,22^. For clinical purposes, TMB is just one biomarker of immunotherapy benefit; these results do not necessarily imply that TMB is predictive for each individual patient or cancer type within the cohort^23^.

In conclusion, we combined limited manual clinical annotation with deep neural networks to complete clinical outcomes extraction for a multi-cancer single-institution clinico-genomic cohort. The resulting annotations reliably captured clinical outcomes, even for cancer types with no labeled data available for training. These outcomes further demonstrated expected patterns of association with overall survival. Analysis of a multi-cancer clinico-genomic dataset generated with this approach confirmed an expected association between tumor mutation burden and progression-free survival on immune checkpoint inhibitor therapy. This approach reduces the barrier posed by clinical annotation tasks to optimal utilization of genomic data to pursue patient-relevant research questions.

## Methods

### Data sources

The cohort for this analysis consisted of patients with solid tumors that had undergone next-generation sequencing at Brigham and Women’s Hospital or Dana-Farber Cancer Institute with the in-house OncoPanel assay from January 1, 2013 to January 31, 2021, and who had at least one imaging report and/or medical oncologist note in the electronic health record^4,10^. Cancer types were selected a priori to focus on a variety of common solid tumors; primary tumor types included breast, colorectal, endometrial, gastric/esophageal, head and neck, leiomyoscarcoma, non-small cell lung, melanoma, high-grade serous ovarian, pancreatic, prostate, renal cell, and urothelial carcinoma. Using the Oncology Data Retrieval System^24^, structured and unstructured electronic health records data for this cohort were obtained. Structured data included somatic genomic mutation calls, including a derived tumor mutation burden; vital status obtained from clinical records and linkage to the National Death Index; diagnosis codes; and systemic therapy treatment plans, including intent of therapy as recorded by treating oncologists. Unstuctured data included clinical progress notes and imaging reports. The analysis was conducted under a waiver of informed consent from the Dana-Farber/Harvard Cancer Center Institutional Review Board given the minimal risk of this retrospective study.

### Manual medical record annotation

The subset of patients whose records underwent manual annotation was derived from separate retrospective cohort studies at DFCI that used the PRISSMM annotation framework. For patients with non-small cell lung, breast, colorectal, or prostate cancer, patients were selected for annotation as part of the American Association for Cancer Research’s Genomics Evidence Neoplasia Information Exchange (Project GENIE)^25^ Biopharmaceutical Consortium. For patients with renal cell carcinoma or urothelial carcinoma, records were annotated by the Dana-Farber Cancer Institute’s Division of Genitourinary Malignancies. The annotation process and calculations of inter-rater reliability have been described previously^6–9^. Briefly, for each imaging report and medical oncologist note, annotators recorded the presence or absence of cancer in that individual document. When cancer was determined to be present, annotators further recorded whether the cancer burden was changing, including whether it was improving/responding, worsening/progressing, stable, mixed, or indeterminate. For purposes of this study, the improving/responding and worsening/progressing endpoints were each treated as two binary variables, such that a “mixed” status was considered neither worsening nor improving. For imaging reports, when cancer was determined to be present, annotators were asked to record specific sites of disease, including brain, bone, adrenal, liver, lung, lymph node, and peritoneum.

### NLP model training and evaluation

Manually labeled records were divided, at the patient level, into training (80%), validation (10%), and test (10%) subsets. Training and inference were performed on a per-document basis. The overall approach to training has been described previously for patients with non-small cell lung cancer^8,9^. Briefly, a convolutional neural network architecture was applied to the text for each document. Distinct models were trained for each outcome; for imaging reports, these included “any cancer,” progression/worsening, response/improvement, and the presence of disease in brian, bone, adrenal liver, lung, lymph node, or peritoneum. For medical oncologist notes, these included “any cancer”, progression/worsening, and response/improvement. The “any cancer” model had a single output, and all other models had two outputs, one for the presence of any cancer, and the other for the specific output of interest. Cross-validation was applied as follows. The training set was divided into 10 subsets, and each model was trained repeatedly on 9 of the 10 subsets. For inference within the training set, each patient’s records were annotated by the models that excluded that patient from training. For inference within the validation and test sets of labeled data, and for application to patients with no labeled data, an ensemble model was applied; the ensemble was created for each outcome by mean-pooling the linear outputs from each of the 10 trained models. The raw output of each model corresponded to the log odds that a given outcome was present in a particular document. For medical oncologist notes, an additional preprocessing step was applied to extract the “assessment/plan” section from each note for input into the convolutional neural network. This preprocessing step consisted of a recurrent neural network trained to identify the words in each note that belonged to the assessment/plan section using a rules-based labeling strategy, as previously described^9^.

To evaluate the generalizability of this approach to cancer types for which no labeled data were available for training, the models for each outcome were repeatedly re-trained after exclusion of patients with each specific type of cancer for which we had labeled data. Inference was then performed, and models evaluated, for the cancer type that had been excluded from training, simulating a situation in which there were no labeled training data for that cancer type.

Models were evaluated initially and hyperparameters manually tuned in the validation set. The primary evaluation metric was the area under the receiver operating characteristic (AUROC) curve. After final models were trained, performance was evaluated in the held-out test set for reporting. Areas under the precision-recall curve and calibration curves were also calculated. Sensitivity, specificity, positive predictive value, and negative predictive value at the best F1 score and best Youden’s index^26^ thresholds for each outcome were also derived. These evaluation metrics are presented in Supplementary Fig. 1A–M.

Models were trained and applied using Tensorflow^27^ version 2.4.1. Code for model training and evaluation is available at github.com/prissmmnlp/pan_cancer_outcomes.

### Evaluation of associations between PRISSMM outcomes and overall survival among patients receiving palliative-intent therapy

To evaluate the face validity of NLP model-curated endpoints for capturing clinically important outcomes associated with survival and further evaluate the generalizability of the endpoints to cancer types in which no labeled data are available, associations were measured between PRISSMM progression/worsening and response/improvement endpoints and overall survival in the subset of the cohort with any palliative-intent systemic therapy treatment plan on record. These analyses were conducted by treating PRISSMM endpoints as time-varying covariates in a Cox proportional hazards model with survival analysis performed using the counting process method^28^. NLP endpoints were derived using cross-validation, such that inference for an individual patient was always performed using models that were not trained on that patient. PRISSMM endpoints were each transformed from continuous NLP model output scores to binary variables by choosing an output threshold that yielded the best F1 (harmonic mean between precision and recall) score. The index date for survival analysis was first palliative systemic therapy, but time intervals beginning prior to genomic testing were not considered at risk, since genomic testing was an eligibility criterion for this analysis and mortality events were therefore not possible prior to testing. Separate multivariable Cox models were constructed to analyze associations between imaging report annotations and OS, and between oncologist note annotations and OS. The principal analyses included patients with all cancer types and further adjusted for cancer type; analyses were also performed separately for patients with each individual cancer type. Hazard ratios and 95% confidence intervals (CIs) associated with progression/worsening and response/improvement were calculated. Analyses were performed using R, version 4.0.3.

### Evaluation of correlations between model-derived progression-free survival endpoints and overall survival

For patients who initiated palliative-intent systemic therapy prior to the end of survival follow-up on December 31, 2019, progression-free survival (PFS) endpoints were derived as previously described^7^, using model-derived progression defined using the best F1 threshold output. Candidate endpoints included PFS-I, or the time to first worsening/progression documented on imaging report, or death; PFS-M, the time to first worsening/progression documented on medical oncologist assessment, or death; PFS-I-or-M, time to first indication of worsening/progression on imaging report or medical oncologist assessment, or death, whichever was earliest; and PFS-I-and-M, time from treatment start to worsening/progression having been documented on both an imaging report and a medical oncologist assessment, or death. Calculations were performed using R (version 4.0.1) and the SurvCorr R package (version 1.0)^29^. Left truncation at the time of genomic testing was not performed for these analyses, since methods for calculating correlations between left-truncated time-to-event endpoints are not well-characterized; however, in prior work we found that restricting analyses to patients initiating therapy after genomic testing did not change calculated correlation coefficients for manually-defined PFS endpoints for patients with NSCLC or colorectal cancer^7^.

### Association between tumor mutation burden and PFS among patients receiving immunotherapy

We identified patients with any of our cancer types who received palliative-intent immune checkpoint inhibitor systemic therapy regimens, defined as treatment that included ipilimumab, nivolumab, pembrolizumamb, atezolizumab, durvalumab, and/or avelumab. Some patients received more than one such regimen. We conducted a time-to-event analysis using Cox proportional hazards modeling. Statistical tests were two-sided. The index time corresponded to initiation of each immunotherapy regimen, and events were defined as PFS-I-and-M, described above. Covariates in each model included tumor mutation burden, derived for each patient using his/her first tumor sample with NGS results^30,31^ and obtained from the Oncology Data Retrieval System at DFCI^24^; cancer type; and line of therapy, accounting for clustering of observations within patients who received more than one line of immunotherapy. Analyses were performed in which TMB was treated as a continuous variable and in which it was dichotomized into high (>=20 mutations/megabase) or low (<20 mutations/megabase). Unadjusted Kaplan–Meier curves for patients with TMB-high tumors and those with TMB-low tumors were also generated. Median PFS and OS in the TMB-high and TMB-low groups were also calculated. For calculation of OS, since genomic testing was a cohort eligibility criterion and mortality events prior to genomic testing were therefore not possible, left truncation was performed at the time of genomic testing using the counting process method^18^. Analyses were performed using R, version 4.0.3.

### Reporting summary

Further information on research design is available in the Nature Research Reporting Summary linked to this article.

## Supplementary information

Supplementary Information

Reporting Summary

## Data Availability

The underlying EHR text reports used to train and evaluate NLP models for these analyses constitute protected health information for DFCI patients and therefore cannot be made publicly available. Researchers with DFCI appointments and Institutional Review Board (IRB) approval can access the data on request. For external researchers, access would require collaboration with the authors and eligibility for a DFCI appointment per DFCI policies. A derived analytic dataset used to examine the association between TMB and PFS among patients receiving immunotherapy has been uploaded to the project Github page (https://github.com/prissmmnlp/pan_cancer_outcomes)^32^. Deidentified genomic data are available for DFCI patients through AACR’s Project GENIE. Deidentified clinical data corresponding to PRISSMM annotations for DFCI patients will be made publicly available on cBioPortal through the AACR Project GENIE Biopharmaceutical Consortium according to a predefined staggered release schedule through the end of 2023.

## Code availability

Code for model training and evaluation is available at github.com/prissmmnlp/pan_cancer_outcomes^32^.
