# Supplementary material for: Artificial intelligence-aided clinical annotation of a large multi-cancer genomic dataset
Source: Nat Commun. 2021 Dec 15;12:7304. doi: 10.1038/s41467-021-27358-6 (PMC8674229; doi:10.1038/s41467-021-27358-6)

Supplementary Table 1: Sensitivity analysis performed to evaluate generalizability of NLP models to reports from a future time period. Areas under the receiver operating characteristic curve (AUROCs) for NLP models that interpret report text to ascertain clinical outcomes, as evaluated in the labeled test set. All models for this analysis were re-trained using reports through 2017.

| Clinical outcome | Imaging reports | | Medical oncologist notes | |
| --- | --- | --- | --- | --- |
|  | Evaluation period: Through 2017 | Evaluation period: 2018 | Evaluation period: Through 2017 | Evaluation period:  2018 |
| Any cancer | 0.97 | 0.98 | 0.92 | 0.93 |
| Progression | 0.94 | 0.96 | 0.92 | 0.90 |
| Response | 0.97 | 0.97 | 0.94 | 0.90 |
| Brain metastasis | 0.99 | 0.99 | † | † |
| Bone metastasis | 0.99 | 0.98 | † | † |
| Adrenal metastasis | 0.99 | 1.0 | † | † |
| Liver metastasis | 0.99 | 0.99 | † | † |
| Lung metastasis | 0.98 | 0.98 | † | † |
| Nodal metastasis | 0.98 | 0.98 | † | † |
| Peritoneal metastasis | 0.99 | 0.99 | † | † |

† Not applicable

Supplementary Figure 1: Additional metrics of model performance

Supplementary Figure 1A: Imaging report model performance, any-cancer outcome, test set

| Metrics at best F1 threshold probability | | | | |
| --- | --- | --- | --- | --- |
|  |  |  | |  |
|  |  | Manual label | | Total |
|  |  | No | Yes |  |
| Predicted label | No | 1087 | 152 | 1239 |
|  | Yes | 80 | 1541 | 1621 |
|  |  |  |  |  |
|  | Total | 1167 | 1693 | 2860 |
|  | | | | |
| Sensitivity | | 91% | | |
| Specificity | | 93% | | |
| Positive predictive value | | 95% | | |
| Negative predictive value | | 88% | | |


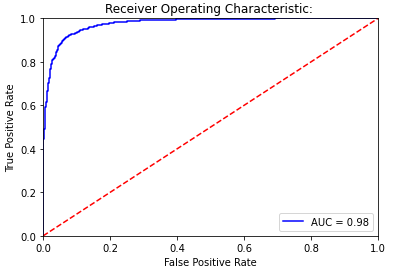


| Metrics at best Youden threshold probability | | | | |
| --- | --- | --- | --- | --- |
|  |  |  | |  |
|  |  | Manual label | | Total |
|  |  | No | Yes |  |
| Predicted label | No | 1089 | 153 | 1242 |
|  | Yes | 78 | 1540 | 1618 |
|  |  |  |  |  |
|  | Total | 1167 | 1693 | 2860 |
|  | | | | |
| Sensitivity | | 91% | | |
| Specificity | | 93% | | |
| Positive predictive value | | 95% | | |
| Negative predictive value | | 88% | | |


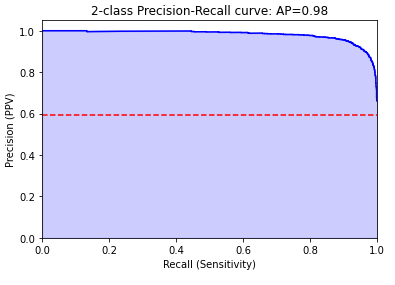


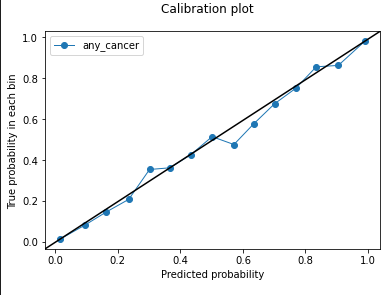


* Red dotted lines in top two panels represent the value of each performance metric that would be expected for an uninformative model. For the area under the receiver operating characteristic curve, this corresponds to a value of 0.5; for the area under the precision-recall curve, this corresponds to the prevalence of the outcome among all reports.

Supplementary Figure 1B: Imaging report model performance, progression outcome, test set

| Metrics at best F1 threshold probability | | | | |
| --- | --- | --- | --- | --- |
|  |  |  | |  |
|  |  | Manual label | | Total |
|  |  | No | Yes |  |
| Predicted label | No | 2052 | 119 | 2171 |
|  | Yes | 155 | 534 | 689 |
|  |  |  |  |  |
|  | Total | 2207 | 653 | 2860 |
|  | | | | |
| Sensitivity | | 82% | | |
| Specificity | | 93% | | |
| Positive predictive value | | 78% | | |
| Negative predictive value | | 95% | | |


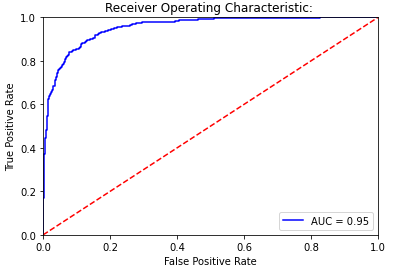


| Metrics at best Youden threshold probability | | | | |
| --- | --- | --- | --- | --- |
|  |  |  | |  |
|  |  | Manual label | | Total |
|  |  | No | Yes |  |
| Predicted label | No | 1930 | 73 | 2003 |
|  | Yes | 277 | 580 | 857 |
|  |  |  |  |  |
|  | Total | 2207 | 653 | 2860 |
|  | | | | |
| Sensitivity | | 89% | | |
| Specificity | | 87% | | |
| Positive predictive value | | 68% | | |
| Negative predictive value | | 96% | | |


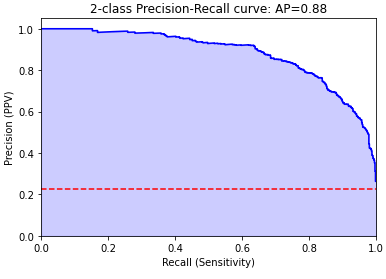


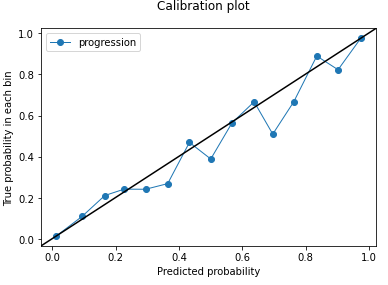


* Red dotted lines in top two panels represent the value of each performance metric that would be expected for an uninformative model. For the area under the receiver operating characteristic curve, this corresponds to a value of 0.5; for the area under the precision-recall curve, this corresponds to the prevalence of the outcome among all reports.

Supplementary Figure 1C: Imaging report model performance, response outcome, test set

| Metrics at best F1 threshold probability | | | | |
| --- | --- | --- | --- | --- |
|  |  |  | |  |
|  |  | Manual label | | Total |
|  |  | No | Yes |  |
| Predicted label | No | 2629 | 55 | 2684 |
|  | Yes | 46 | 130 | 176 |
|  |  |  |  |  |
|  | Total | 2675 | 185 | 2860 |
|  | | | | |
| Sensitivity | | 70% | | |
| Specificity | | 98% | | |
| Positive predictive value | | 74% | | |
| Negative predictive value | | 98% | | |


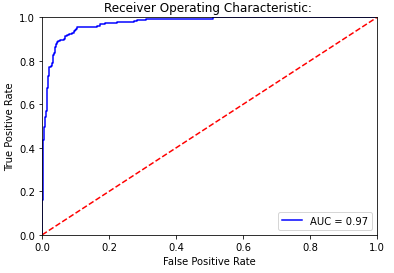


| Metrics at best Youden threshold probability | | | | |
| --- | --- | --- | --- | --- |
|  |  |  | |  |
|  |  | Manual label | | Total |
|  |  | No | Yes |  |
| Predicted label | No | 2453 | 14 | 2467 |
|  | Yes | 222 | 171 | 393 |
|  |  |  |  |  |
|  | Total | 2675 | 171 | 393 |
|  | | | | |
| Sensitivity | | 92% | | |
| Specificity | | 92% | | |
| Positive predictive value | | 44% | | |
| Negative predictive value | | 99% | | |


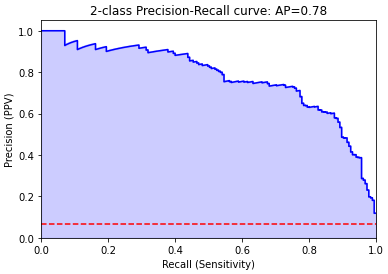


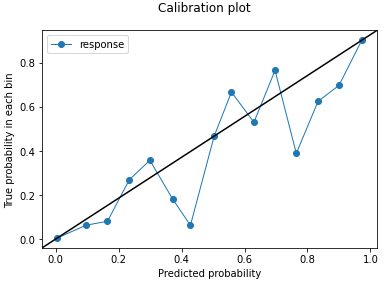


* Red dotted lines in top two panels represent the value of each performance metric that would be expected for an uninformative model. For the area under the receiver operating characteristic curve, this corresponds to a value of 0.5; for the area under the precision-recall curve, this corresponds to the prevalence of the outcome among all reports.

Supplementary Figure 1D: Imaging report model performance, brain metastasis outcome, test set

| Metrics at best F1 threshold probability | | | | |
| --- | --- | --- | --- | --- |
|  |  |  | |  |
|  |  | Manual label | | Total |
|  |  | No | Yes |  |
| Predicted label | No | 2595 | 25 | 2620 |
|  | Yes | 42 | 198 | 240 |
|  |  |  |  |  |
|  | Total | 2637 | 223 | 2860 |
|  | | | | |
| Sensitivity | | 89% | | |
| Specificity | | 98% | | |
| Positive predictive value | | 83% | | |
| Negative predictive value | | 99% | | |


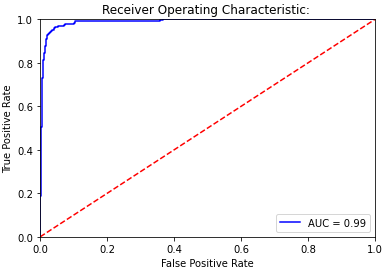


| Metrics at best Youden threshold probability | | | | |
| --- | --- | --- | --- | --- |
|  |  |  | |  |
|  |  | Manual label | | Total |
|  |  | No | Yes |  |
| Predicted label | No | 2509 | 8 | 2517 |
|  | Yes | 128 | 215 | 343 |
|  |  |  |  |  |
|  | Total | 2637 | 223 | 2860 |
|  | | | | |
| Sensitivity | | 96% | | |
| Specificity | | 95% | | |
| Positive predictive value | | 63% | | |
| Negative predictive value | | 99.7% | | |


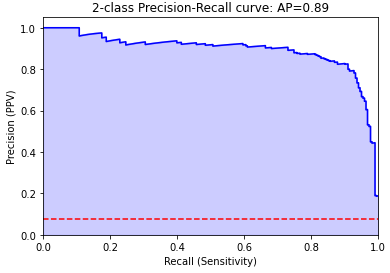


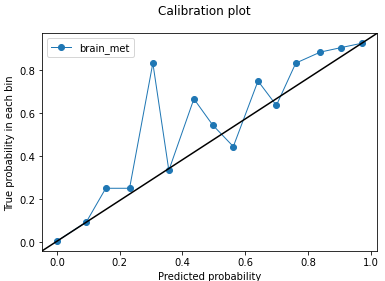


* Red dotted lines in top two panels represent the value of each performance metric that would be expected for an uninformative model. For the area under the receiver operating characteristic curve, this corresponds to a value of 0.5; for the area under the precision-recall curve, this corresponds to the prevalence of the outcome among all reports.

Supplementary Figure 1E: Imaging report model performance, lung metastasis outcome, test set

| Metrics at best F1 threshold probability | | | | |
| --- | --- | --- | --- | --- |
|  |  |  | |  |
|  |  | Manual label | | Total |
|  |  | No | Yes |  |
| Predicted label | No | 2251 | 50 | 2301 |
|  | Yes | 121 | 438 | 559 |
|  |  |  |  |  |
|  | Total | 2372 | 488 | 2860 |
|  | | | | |
| Sensitivity | | 90% | | |
| Specificity | | 95% | | |
| Positive predictive value | | 78% | | |
| Negative predictive value | | 98% | | |


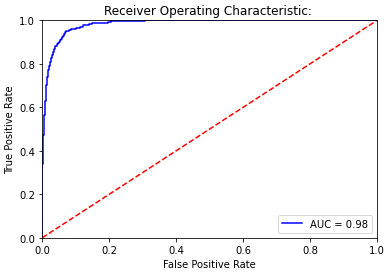


| Metrics at best Youden threshold probability | | | | |
| --- | --- | --- | --- | --- |
|  |  |  | |  |
|  |  | Manual label | | Total |
|  |  | No | Yes |  |
| Predicted label | No | 2169 | 21 | 2190 |
|  | Yes | 203 | 467 | 670 |
|  |  |  |  |  |
|  | Total | 2372 | 488 | 2860 |
|  | | | | |
| Sensitivity | | 96% | | |
| Specificity | | 91% | | |
| Positive predictive value | | 70% | | |
| Negative predictive value | | 99% | | |


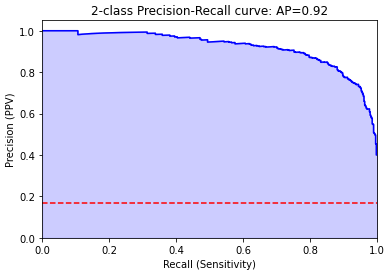


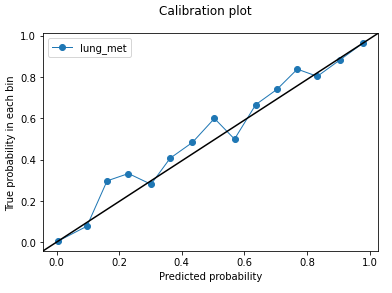


* Red dotted lines in top two panels represent the value of each performance metric that would be expected for an uninformative model. For the area under the receiver operating characteristic curve, this corresponds to a value of 0.5; for the area under the precision-recall curve, this corresponds to the prevalence of the outcome among all reports.

Supplementary Figure 1F: Imaging report model performance, bone metastasis outcome, test set

| Metrics at best F1 threshold probability | | | | |
| --- | --- | --- | --- | --- |
|  |  |  | |  |
|  |  | Manual label | | Total |
|  |  | No | Yes |  |
| Predicted label | No | 2181 | 58 | 2239 |
|  | Yes | 67 | 554 | 621 |
|  |  |  |  |  |
|  | Total | 2248 | 612 | 2860 |
|  | | | | |
| Sensitivity | | 91% | | |
| Specificity | | 97% | | |
| Positive predictive value | | 89% | | |
| Negative predictive value | | 97% | | |


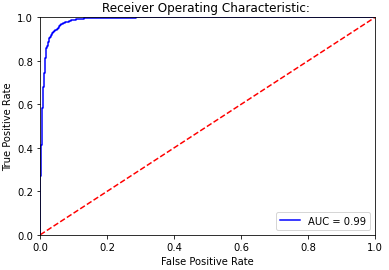


| Metrics at best Youden threshold probability | | | | |
| --- | --- | --- | --- | --- |
|  |  |  | |  |
|  |  | Manual label | | Total |
|  |  | No | Yes |  |
| Predicted label | No | 2105 | 19 | 2124 |
|  | Yes | 143 | 593 | 736 |
|  |  |  |  |  |
|  | Total | 2248 | 612 | 2860 |
|  | | | | |
| Sensitivity | | 97% | | |
| Specificity | | 94% | | |
| Positive predictive value | | 81% | | |
| Negative predictive value | | 99% | | |


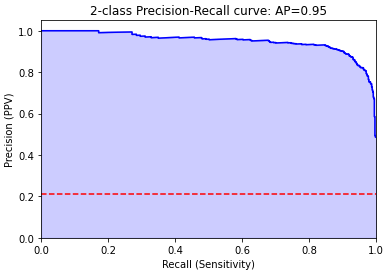


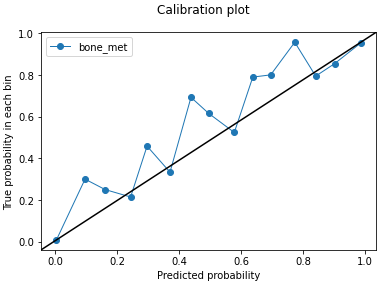


* Red dotted lines in top two panels represent the value of each performance metric that would be expected for an uninformative model. For the area under the receiver operating characteristic curve, this corresponds to a value of 0.5; for the area under the precision-recall curve, this corresponds to the prevalence of the outcome among all reports.

Supplementary Figure 1G: Imaging report model performance, liver metastasis outcome, test set

| Metrics at best F1 threshold probability | | | | |
| --- | --- | --- | --- | --- |
|  |  |  | |  |
|  |  | Manual label | | Total |
|  |  | No | Yes |  |
| Predicted label | No | 2446 | 29 | 2475 |
|  | Yes | 57 | 328 | 385 |
|  |  |  |  |  |
|  | Total | 57 | 328 | 385 |
|  | | | | |
| Sensitivity | | 92% | | |
| Specificity | | 98% | | |
| Positive predictive value | | 86% | | |
| Negative predictive value | | 99% | | |


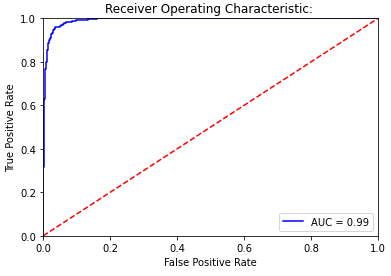


| Metrics at best Youden threshold probability | | | | |
| --- | --- | --- | --- | --- |
|  |  |  | |  |
|  |  | Manual label | | Total |
|  |  | No | Yes |  |
| Predicted label | No | 2364 | 11 | 2375 |
|  | Yes | 139 | 346 | 485 |
|  |  |  |  |  |
|  | Total | 2503 | 357 | 2860 |
|  | | | | |
| Sensitivity | | 97% | | |
| Specificity | | 94% | | |
| Positive predictive value | | 71% | | |
| Negative predictive value | | 99.5% | | |


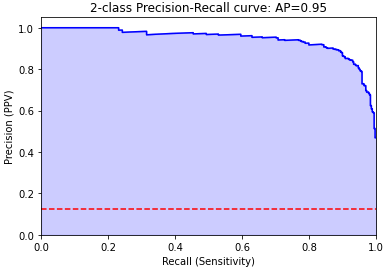


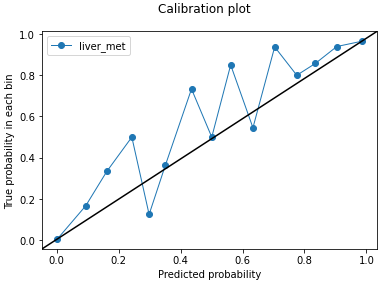


* Red dotted lines in top two panels represent the value of each performance metric that would be expected for an uninformative model. For the area under the receiver operating characteristic curve, this corresponds to a value of 0.5; for the area under the precision-recall curve, this corresponds to the prevalence of the outcome among all reports.

Supplementary Figure 1H: Imaging report model performance, adrenal metastasis outcome, test set

| Metrics at best F1 threshold probability | | | | |
| --- | --- | --- | --- | --- |
|  |  |  | |  |
|  |  | Manual label | | Total |
|  |  | No | Yes |  |
| Predicted label | No | 2734 | 6 | 2740 |
|  | Yes | 51 | 69 | 120 |
|  |  |  |  |  |
|  | Total | 2785 | 75 | 2860 |
|  | | | | |
| Sensitivity | | 92% | | |
| Specificity | | 98% | | |
| Positive predictive value | | 58% | | |
| Negative predictive value | | 99.7% | | |


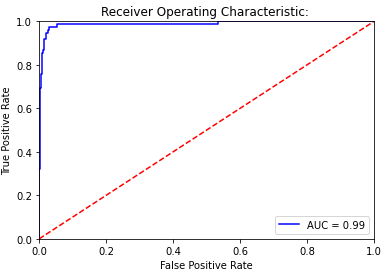


| Metrics at best Youden threshold probability | | | | |
| --- | --- | --- | --- | --- |
|  |  |  | |  |
|  |  | Manual label | | Total |
|  |  | No | Yes |  |
| Predicted label | No | 2682 | 2 | 2684 |
|  | Yes | 103 | 73 | 176 |
|  |  |  |  |  |
|  | Total | 2785 | 75 | 2860 |
|  | | | | |
| Sensitivity | | 97% | | |
| Specificity | | 96% | | |
| Positive predictive value | | 41% | | |
| Negative predictive value | | 99.9% | | |


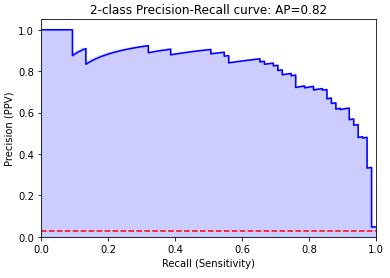


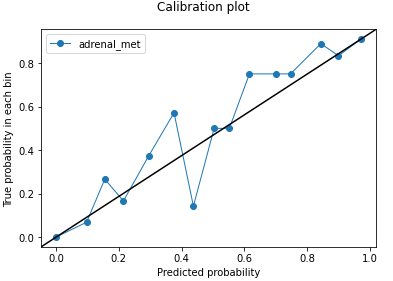


* Red dotted lines in top two panels represent the value of each performance metric that would be expected for an uninformative model. For the area under the receiver operating characteristic curve, this corresponds to a value of 0.5; for the area under the precision-recall curve, this corresponds to the prevalence of the outcome among all reports.

Supplementary Figure 1I: Imaging report model performance, lymph node metastasis outcome, test set

| Metrics at best F1 threshold probability | | | | |
| --- | --- | --- | --- | --- |
|  |  |  | |  |
|  |  | Manual label | | Total |
|  |  | No | Yes |  |
| Predicted label | No | 2470 | 80 | 2550 |
|  | Yes | 51 | 259 | 310 |
|  |  |  |  |  |
|  | Total | 2521 | 339 | 2860 |
|  | | | | |
| Sensitivity | | 76% | | |
| Specificity | | 98% | | |
| Positive predictive value | | 84% | | |
| Negative predictive value | | 97% | | |


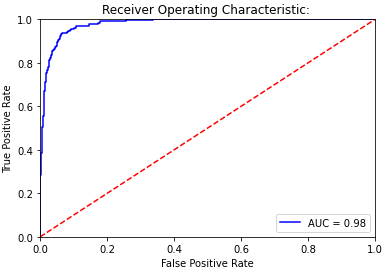


| Metrics at best Youden threshold probability | | | | |
| --- | --- | --- | --- | --- |
|  |  |  | |  |
|  |  | Manual label | | Total |
|  |  | No | Yes |  |
| Predicted label | No | 2350 | 22 | 2372 |
|  | Yes | 171 | 317 | 488 |
|  |  |  |  |  |
|  | Total | 2521 | 339 | 2860 |
|  | | | | |
| Sensitivity | | 94% | | |
| Specificity | | 93% | | |
| Positive predictive value | | 65% | | |
| Negative predictive value | | 99% | | |


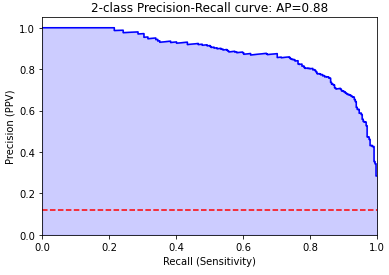


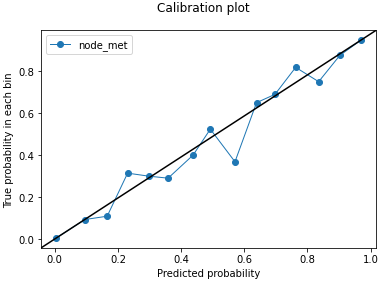


* Red dotted lines in top two panels represent the value of each performance metric that would be expected for an uninformative model. For the area under the receiver operating characteristic curve, this corresponds to a value of 0.5; for the area under the precision-recall curve, this corresponds to the prevalence of the outcome among all reports.

Supplementary Figure 1J: Imaging report model performance, peritoneal metastasis outcome, test set

| Metrics at best F1 threshold probability | | | | |
| --- | --- | --- | --- | --- |
|  |  |  | |  |
|  |  | Manual label | | Total |
|  |  | No | Yes |  |
| Predicted label | No | 2752 | 17 | 2769 |
|  | Yes | 19 | 72 | 91 |
|  |  |  |  |  |
|  | Total | 2771 | 89 | 2860 |
|  | | | | |
| Sensitivity | | 81% | | |
| Specificity | | 99% | | |
| Positive predictive value | | 79% | | |
| Negative predictive value | | 99% | | |


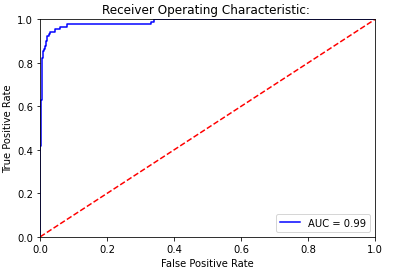


| Metrics at best Youden threshold probability | | | | |
| --- | --- | --- | --- | --- |
|  |  |  | |  |
|  |  | Manual label | | Total |
|  |  | No | Yes |  |
| Predicted label | No | 2688 | 5 | 2693 |
|  | Yes | 83 | 84 | 167 |
|  |  |  |  |  |
|  | Total | 2771 | 89 | 2860 |
|  | | | | |
| Sensitivity | | 94% | | |
| Specificity | | 97% | | |
| Positive predictive value | | 50% | | |
| Negative predictive value | | 99.8% | | |


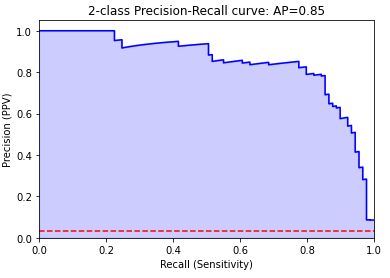


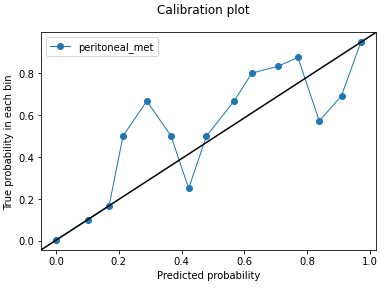


* Red dotted lines in top two panels represent the value of each performance metric that would be expected for an uninformative model. For the area under the receiver operating characteristic curve, this corresponds to a value of 0.5; for the area under the precision-recall curve, this corresponds to the prevalence of the outcome among all reports.

Supplementary Figure 1K: Medical oncologist note model performance, any cancer outcome, test set

| Metrics at best F1 threshold probability | | | | |
| --- | --- | --- | --- | --- |
|  |  |  | |  |
|  |  | Manual label | | Total |
|  |  | No | Yes |  |
| Predicted label | No | 370 | 83 | 453 |
|  | Yes | 184 | 2156 | 2340 |
|  |  |  |  |  |
|  | Total | 554 | 2239 | 2793 |
|  | | | | |
| Sensitivity | | 96% | | |
| Specificity | | 67% | | |
| Positive predictive value | | 92% | | |
| Negative predictive value | | 82% | | |


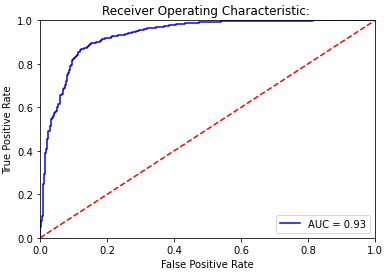


| Metrics at best Youden threshold probability | | | | |
| --- | --- | --- | --- | --- |
|  |  |  | |  |
|  |  | Manual label | | Total |
|  |  | No | Yes |  |
| Predicted label | No | 449 | 199 | 648 |
|  | Yes | 105 | 2040 | 2145 |
|  |  |  |  |  |
|  | Total | 554 | 2239 | 2793 |
|  | | | | |
| Sensitivity | | 91% | | |
| Specificity | | 81% | | |
| Positive predictive value | | 95% | | |
| Negative predictive value | | 69% | | |


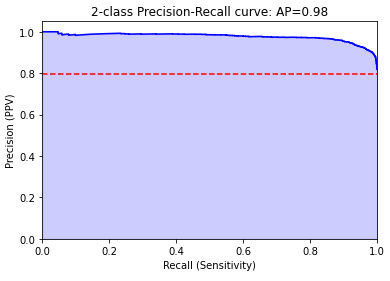


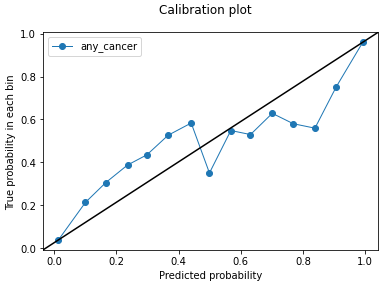


* Red dotted lines in top two panels represent the value of each performance metric that would be expected for an uninformative model. For the area under the receiver operating characteristic curve, this corresponds to a value of 0.5; for the area under the precision-recall curve, this corresponds to the prevalence of the outcome among all reports.

Supplementary Figure 1L: Medical oncologist note model performance, progression outcome, test set

| Metrics at best F1 threshold probability | | | | |
| --- | --- | --- | --- | --- |
|  |  |  | |  |
|  |  | Manual label | | Total |
|  |  | No | Yes |  |
| Predicted label | No | 2102 | 95 | 2197 |
|  | Yes | 243 | 353 | 596 |
|  |  |  |  |  |
|  | Total | 2345 | 448 | 2793 |
|  | | | | |
| Sensitivity | | 79% | | |
| Specificity | | 90% | | |
| Positive predictive value | | 59% | | |
| Negative predictive value | | 96% | | |


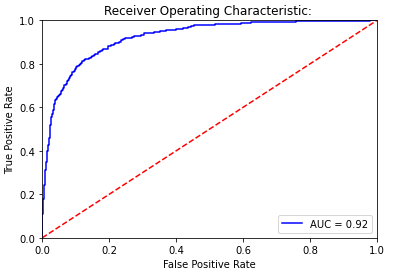


| Metrics at best Youden threshold probability | | | | |
| --- | --- | --- | --- | --- |
|  |  |  | |  |
|  |  | Manual label | | Total |
|  |  | No | Yes |  |
| Predicted label | No | 2013 | 79 | 2092 |
|  | Yes | 332 | 369 | 701 |
|  |  |  |  |  |
|  | Total | 2345 | 448 | 2793 |
|  | | | | |
| Sensitivity | | 82% | | |
| Specificity | | 86% | | |
| Positive predictive value | | 53% | | |
| Negative predictive value | | 96% | | |


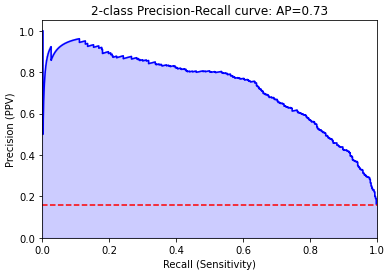


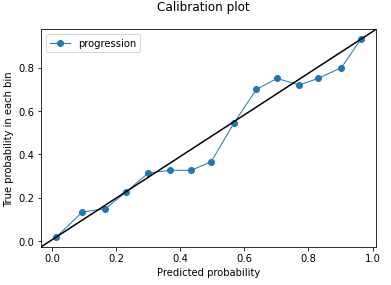


* Red dotted lines in top two panels represent the value of each performance metric that would be expected for an uninformative model. For the area under the receiver operating characteristic curve, this corresponds to a value of 0.5; for the area under the precision-recall curve, this corresponds to the prevalence of the outcome among all reports.

Supplementary Figure 1M: Medical oncologist note model performance, response outcome, test set

| Metrics at best F1 threshold probability | | | | |
| --- | --- | --- | --- | --- |
|  |  |  | |  |
|  |  | Manual label | | Total |
|  |  | No | Yes |  |
| Predicted label | No | 2424 | 113 | 2537 |
|  | Yes | 68 | 188 | 256 |
|  |  |  |  |  |
|  | Total | 2492 | 301 | 2793 |
|  | | | | |
| Sensitivity | | 62% | | |
| Specificity | | 97% | | |
| Positive predictive value | | 73% | | |
| Negative predictive value | | 96% | | |


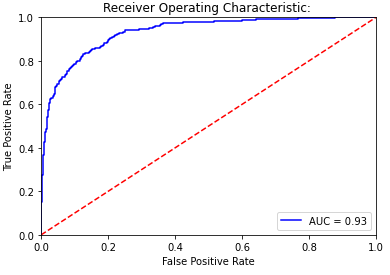


| Metrics at best Youden threshold probability | | | | |
| --- | --- | --- | --- | --- |
|  |  |  | |  |
|  |  | Manual label | | Total |
|  |  | No | Yes |  |
| Predicted label | No | 2224 | 62 | 2286 |
|  | Yes | 268 | 239 | 507 |
|  |  |  |  |  |
|  | Total | 2492 | 301 | 2793 |
|  | | | | |
| Sensitivity | | 79% | | |
| Specificity | | 89% | | |
| Positive predictive value | | 47% | | |
| Negative predictive value | | 97% | | |


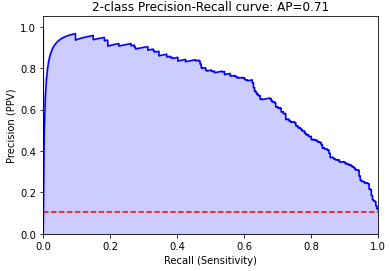


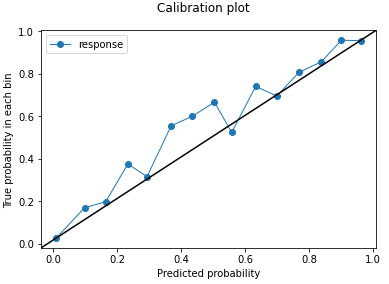


* Red dotted lines in top two panels represent the value of each performance metric that would be expected for an uninformative model. For the area under the receiver operating characteristic curve, this corresponds to a value of 0.5; for the area under the precision-recall curve, this corresponds to the prevalence of the outcome among all reports.

Supplementary Figure 2: Kaplan-Meier curves for time to event outcomes derived using natural processing models after initiation of first-line palliative-intent systemic therapy.


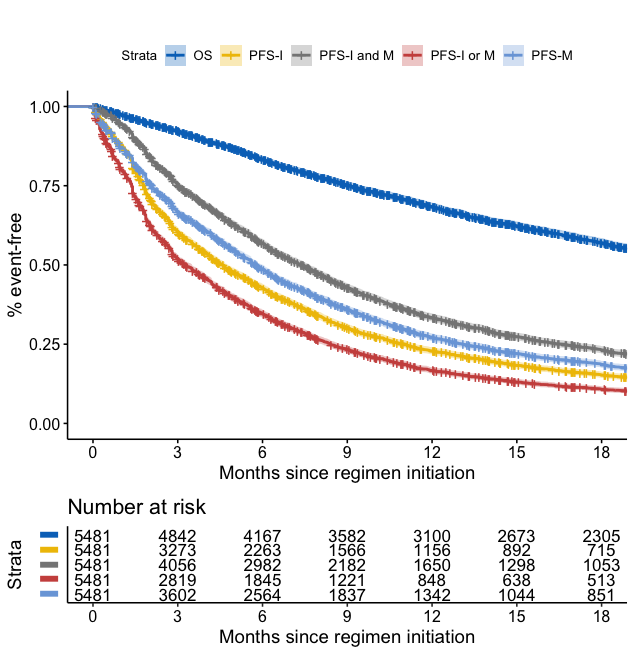


OS, overall survival; PFS-I, progression-free survival with progression defined by imaging reports; PFS-M, progression free survival with progression defined by medical oncology assessment; PFS-I-or-M, progression free survival with progression defined by either imaging reports or medical oncologist assessment, whichever came first; PFS-I-and-M, progression free survival with progression defined by the latter of progression by imaging or progression by medical oncologist assessment.

Supplementary Figure 3A: Example of a clinico-genomic analysis based on outcomes ascertained using natural language processing models: Association between TMB and progression-free survival after initation of immunotherapy (labeled cancer types only, including breast cancer, colorectal cancer, non-small cell lung cancer, pancreatic cancer, prostate cancer, renal cell carcinoma, and urothelial cancer)


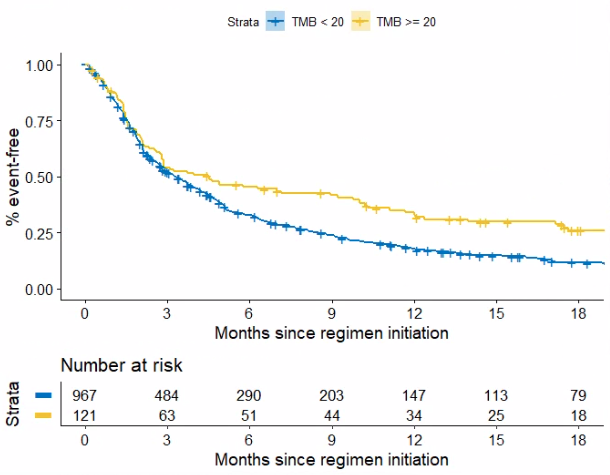


Supplementary Figure 3B: Example of a clinico-genomic analysis based on outcomes ascertained using natural language processing models: Association between TMB and progression-free survival after initation of immunotherapy (unlabeled cancer types only, including endometrial cancer, gastroesophageal cancer, head and neck cancer, high-grade serous ovarian cancer, leiomyosarcoma, melanoma).


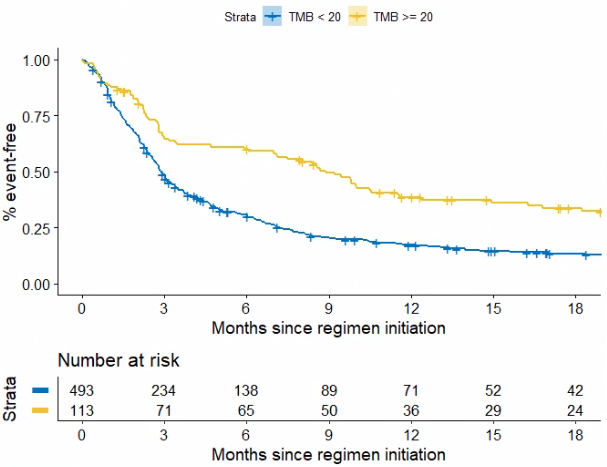

Supplement: Supplementary file 1 — Supplementary Information [file 41467_2021_27358_MOESM1_ESM.docx]
