# Supplementary material for: Artificial intelligence-aided clinical annotation of a large multi-cancer genomic dataset
Source: Nat Commun. 2021 Dec 15;12:7304. doi: 10.1038/s41467-021-27358-6 (PMC8674229; doi:10.1038/s41467-021-27358-6)
Supplement: Supplementary file 2 — Reporting Summary [file 41467_2021_27358_MOESM2_ESM.pdf]

nature portfolio

Corresponding author(s): Kenneth L. Kehl

Last updated by author(s): November 6, 2021

Reporting Summary

Nature Portfolio wishes to improve the reproducibility of the work that we publish. This form provides structure for consistency and transparency in reporting. For further information on Nature Portfolio policies, see our Editorial Policies and the Editorial Policy Checklist.

Statistics

For all statistical analyses, confirm that the following items are present in the figure legend, table legend, main text, or Methods section.

n/a	Confirmed	
☐	☒	The exact sample size (n) for each experimental group/condition, given as a discrete number and unit of measurement
☐	☒	A statement on whether measurements were taken from distinct samples or whether the same sample was measured repeatedly
☐	☒	The statistical test(s) used AND whether they are one- or two-sided<br>*Only common tests should be described solely by name; describe more complex techniques in the Methods section.*
☐	☒	A description of all covariates tested
☒	☐	A description of any assumptions or corrections, such as tests of normality and adjustment for multiple comparisons
☐	☒	A full description of the statistical parameters including central tendency (e.g. means) or other basic estimates (e.g. regression coefficient) AND variation (e.g. standard deviation) or associated estimates of uncertainty (e.g. confidence intervals)
☐	☒	For null hypothesis testing, the test statistic (e.g. F, t, r) with confidence intervals, effect sizes, degrees of freedom and P value noted<br>*Give P values as exact values whenever suitable.*
☒	☐	For Bayesian analysis, information on the choice of priors and Markov chain Monte Carlo settings
☒	☐	For hierarchical and complex designs, identification of the appropriate level for tests and full reporting of outcomes
☒	☐	Estimates of effect sizes (e.g. Cohen's d, Pearson's r), indicating how they were calculated

Our web collection on statistics for biologists contains articles on many of the points above.

Software and code

Policy information about availability of computer code

Data collection	Clinical data were obtained from the DFCI OncDRS system; code was not used to collect the data per se.
Data analysis	Model training and evaluation code is available at github.com/prissmmnlp/pan_cancer_outcomes , as described in the Methods. Code was written using open source software packages, including Tensorflow (version 2.4.1); and the SurvCorr R package (version 1.0); as described in the Methods.

For manuscripts utilizing custom algorithms or software that are central to the research but not yet described in published literature, software must be made available to editors and reviewers. We strongly encourage code deposition in a community repository (e.g. GitHub). See the Nature Portfolio guidelines for submitting code & software for further information.

Data

Policy information about availability of data

All manuscripts must include a data availability statement. This statement should provide the following information, where applicable:

- Accession codes, unique identifiers, or web links for publicly available datasets
- A description of any restrictions on data availability
- For clinical datasets or third party data, please ensure that the statement adheres to our policy

The underlying EHR text data used to train and evaluate NLP models for these analyses constitute protected health information and as such can not be made publicly available, but derived manually deidentified data used for downstream analyses in this paper can be provided by the corresponding author on reasonable request. Deidentified genomic data are available for DFCI patients through AACR's Project GENIE (https://genie.cbioportal.org/study/summary?id=genie_public). Deidentified clinical data corresponding to PRISSMM annotations for DFCI patients will be made publicly available on cBioPortal through the AACR Project GENIE Biopharmaceutical Consortium according to a predefined staggered release schedule through the end of 2023.

Field-specific reporting

Please select the one below that is the best fit for your research. If you are not sure, read the appropriate sections before making your selection.

- [x] Life sciences
- [ ] Behavioural & social sciences
- [ ] Ecological, evolutionary & environmental sciences

For a reference copy of the document with all sections, see nature.com/documents/nr-reporting-summary-flat.pdf

Life sciences study design

All studies must disclose on these points even when the disclosure is negative.

Sample size	This was a retrospective cohort study of patients with cancer whose tumors had undergone genomic sequencing at Dana-Farber Cancer Institute. Sample sizes were therefore determined simply by the number of such patients who had sequencing at our institution.
Data exclusions	No data were systematically excluded, except as described by the inclusion criteria in the "Data sources" paragraph of the Online Methods section.
Replication	We split the cohort upfront into training, validation, and test sets; and initial results evaluated in the validation set were confirmed by evaluation in the test set. To avoid training-test set information leakage, the training process was not replicated further after evaluation in the test set.
Randomization	This was not an interventional study; randomization is not applicable.
Blinding	This was not an interventional study; randomization is not applicable.

Reporting for specific materials, systems and methods

We require information from authors about some types of materials, experimental systems and methods used in many studies. Here, indicate whether each material, system or method listed is relevant to your study. If you are not sure if a list item applies to your research, read the appropriate section before selecting a response.

Materials & experimental systems

n/a	Involved in the study	
x		Antibodies
x		Eukaryotic cell lines
x		Palaeontology and archaeology
x		Animals and other organisms
	x	Human research participants
x		Clinical data
x		Dual use research of concern

Methods

n/a	Involved in the study	
x		ChIP-seq
x		Flow cytometry
x		MRI-based neuroimaging

Human research participants

Policy information about studies involving human research participants

Population characteristics	This was not an interventional study; it was determined by the DF/HCC IRB to be a minimal risk retrospective medical record review study. Patient characteristics are provided in Tables 1 and 2.
Recruitment	This was not an interventional study; it was determined by the DF/HCC IRB to be a minimal risk retrospective medical record review study. Patients were not prospectively recruited.
Ethics oversight	The analysis was conducted under a waiver of informed consent from the Dana-Farber/Harvard Cancer Center Institutional Review Board given the minimal risk of this retrospective study.

Note that full information on the approval of the study protocol must also be provided in the manuscript.
